# Importance of early detection of infantile inflammatory bowel disease with defective IL-10 pathway: A case report

**DOI:** 10.1097/MD.0000000000025868

**Published:** 2021-05-28

**Authors:** Hua-Hsi Hung, Hung-Chang Lee, Chun-Yan Yeung, Nien-Lu Wang, Tzu-Yin Tang, Harland S. Winter, Judith R. Kelsen, Chuen-Bin Jiang

**Affiliations:** a Department of Pediatric Gastroenterology, Hepatology and Nutrition, MacKay Children's Hospital, Taipei; b Department of Medicine, MacKay Medical College, New Taipei City; c Department of Pediatric Surgery, MacKay Children's Hospital; d Department of Pathology, MacKay Memorial Hospital, Taipei, Taiwan; e Department of Pediatric Gastroenterology, Hepatology and Nutrition, MassGeneral Hospital for Children, Boston, MA; f Children's Hospital of Philadelphia, Department of Pediatrics, Philadelphia, PA.

**Keywords:** enterocolitis, hematopoietic stem cell transplantation, IL-10 receptor mutation, infantile inflammatory bowel disease, perianal fistula

## Abstract

**Rationale:**

Infantile inflammatory bowel disease (IBD) is an extremely rare subgroup of IBD that includes patients whose age of onset is younger than 2 years old. These patients can have more surgical interventions, and a severe and refractory disease course with higher rates of conventional treatment failure. Monogenic defects play an important role in this subgroup of IBD, and identification of the underlying defect can guide the therapeutic approach.

**Patient concerns:**

In 2007, a 4-month-old girl from a nonconsanguineous family presenting with anal fistula, chronic diarrhea, and failure to thrive. She underwent multiple surgical repairs but continued to have persistent colitis and perianal fistulas.

**Diagnosis:**

Crohn's disease was confirmed by endoscopic and histologic finding.

**Intervention:**

Conventional pediatric IBD therapy including multiple surgical interventions and antitumor necrosis factor alpha agents were applied.

**Outcomes:**

The patient did not respond to conventional pediatric IBD therapy. Interleukin-10 (IL-10) receptor mutation was discovered by whole-exome sequencing and defective IL-10 signaling was proved by functional test of IL-10 signaling pathway by the age of 12. The patient is currently awaiting hematopoietic stem cell transplantation.

**Lessons:**

Early detection of underlying genetic causes of patients with infantile-IBD is crucial, since it may prevent patients from undergoing unnecessary surgeries and adverse effects from ineffective medical therapies. Moreover, infantile-IBD patients with complex perianal disease, intractable early onset enterocolitis and extraintestinal manifestations including oral ulcers and skin folliculitis, should undergo genetic and functional testing for IL-10 pathway defect.

## 1 Introduction

Infantile inflammatory bowel disease (IBD) is an extremely rare subgroup of IBD. The pediatric Paris modification of the Montreal classification defined pediatric patients with age of onset younger than 10 years old as subgroup A1a (early-onset IBD), but recent studies suggested subdividing those with a diagnosis before 6 years of age as very-early-onset IBD (VEO-IBD) and those with a diagnosis before 2 years of age as infantile-IBD based on their unique clinical features.^[1–3]^

Patients with VEO-IBD can experience a more severe disease course that is refractory to conventional therapy and have higher morbidity and mortality rates.^[4]^ Some children with VEO-IBD have an underlying defect responsible for their disease and therefore require a different therapeutic approach as compared to older pediatric and adult patients with IBD. The phenotype of VEO-IBD is heterogeneous, however, there is more exclusive colonic disease at presentation and they more often diagnosed with inflammatory bowel disease-unclassified as compared to other age groups, due to inconclusive pathological findings and known progression of disease.^[3,5]^ Some of these differences are due to the unique etiology of VEO-IBD, including monogenic defects, especially in patients with infantile-IBD. More than 60 genes have been identified to be associated with IBD-like manifestations, including X-linked inhibitor of apoptosis protein, IL-10 receptor (IL-10R), and forkhead box protein P3. Different genetic defects can present with specific intestinal and extraintestinal manifestations (e.g., arthritis, vasculitis, skin folliculitis, hair abnormalities, autoimmune hemolytic anemia, eczema, immune deficiency, etc) and can have different ages of onset.^[6]^

Mutations in IL-10R inducing IL-10 signaling pathway defect were the first monogenic defects identified in children with infantile-IBD.^[7,8]^ Here we report the first case of infantile inflammatory bowel disease with heterozygous mutations in interleukin 10 receptor subunit alpha (IL-10RA) in Taiwan, highlight the initial presentation of this disease and review the up-to-date information of diagnostic and therapeutic approach for infantile-IBD.

## 2 Case report

**Figure 1:**
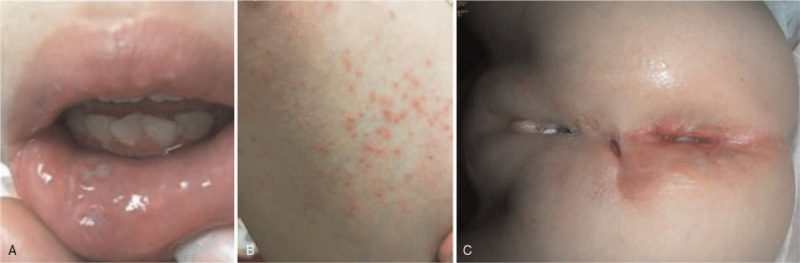
Extraintestinal manifestations of recurrent oral ulcers (A), skin folliculitis (B), and anocutaneous fistulas (C).

In March 2009, a 2-year-old girl was admitted to our hospital with the chief complaint of fever and bloody diarrhea for more than 1 week, growth failure and severe perianal disease since infancy. The pregnancy and delivery were unremarkable, as was the family history. At 4 months of age, the patient developed recurrent perianal abscess, colovaginal fistula and recurrent bacterial enterocolitis, and required multiple surgical interventions. On examination, her weight and height were both below the 3rd percentile. Marked abdominal distention and perineal ulcers with poor-healing and pus-like discharge were noted. In addition, she had multiple oral ulcers and cutaneous folliculitis (Fig. 1). Laboratory workup revealed anemia, leukocytosis, hypoalbuminemia, and a high C-reactive protein level. The immune work up showed normal numbers and function of lymphocytes and neutrophil granulocytes. The complements and serum immunoglobulin levels were within normal limits (data not shown). Abdominal X-ray discovered colon distention and her stool was positive for *Clostridium difficile* glutamate dehydrogenase antigen and toxin. Metronidazole was administered, and a diverting colostomy was performed to treat a presumptive diagnosis of pseudomembranous colitis with toxic megacolon. Pathologic finding revealed nonspecific chronic inflammation. Two weeks after the surgery, an ileostomy was performed to treat an obstruction of the proximal colon.

**Figure 2:**
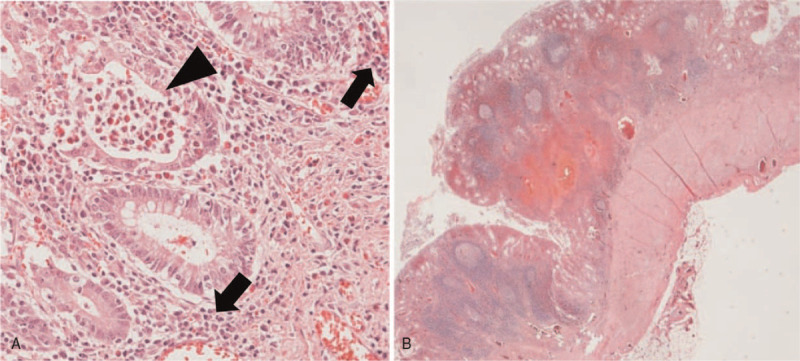
Histological findings of total colectomy. (A) Crypt abscess (arrowhead) and basal lymphoplasmacytosis (arrow) (Hematoxylin and eosin stain, 100×) (B) architectural distortion without transmural involvement. (Hematoxylin and eosin stain, 10×).

**Figure 3:**
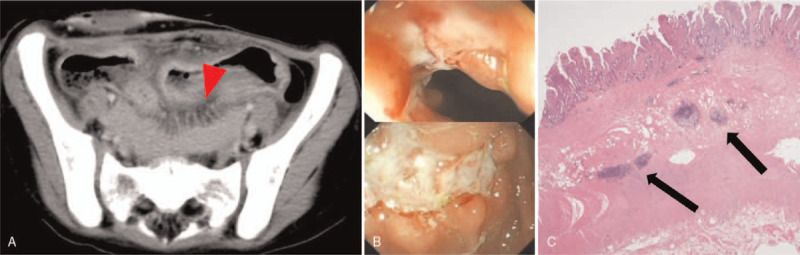
(A) Ileum wall swelling and Comb sign (arrowhead) on contrast-enhanced axial computed tomography scan (B) Ileoscope revealed deep linear ulcerations in ileum. (C) Lymphocytes aggregated in muscularis propia (arrow) on ileal histopathology (Hematoxylin and eosin stain, 10×).

In the next 3 years, multiple attempts to close the ileostomy failed due to recurrent episodes of colitis complicated with perianal ulcerations. Eventually a total colectomy was performed and the histology revealed severe total colitis, basal lympholplasmacytosis without identifiable granuloma and transmural involvement, which were more consistent with ulcerative colitis (UC) (Fig. 2). She was initially treated with corticosteroids and mesalazine, and ileal pouch-anal anastomosis was created. However, severe pouchitis, pouch (ano-perineal-vestibular) fistula and enterocutaneous fistula around the ileostomy developed, raising a concern for Crohn's disease (CD). At 8 years of age, the diagnosis of CD was supported based on a combination of imaging, the endoscopy and ileal histopathology (Fig. 3). Treatment with adalimumab and azathioprine were initiated, and the patient's symptoms partially improved. She continued to have oral ulcers, but had adequate weight gain, and most of the enterocutaneous fistulas healed. Skin folliculitis also subsided. However, she continued to experience multiple exacerbations of her disease, requiring periodic short-term systemic steroid therapies and escalation of adalimumab maintenance dosage. Whole-exome sequencing (WES) identified a missense mutation in IL-10RA (p.Arg101Trp, c.C301T) in 1 allele, but no mutation could be found in the other allele. Functional testing of IL-10 signaling pathway (credited the laboratory in Milwaukee) revealed abnormal suppression of IL-6 secretion in response to lipopolysaccharide/IL-10 co-stimulated peripheral blood mononuclear cell. The result was consistent with an IL-10 receptor defect.

The patient is currently 12 years old, and continues to have poor growth. Her weight and height are 33.6 kg (18th percentile) and 128 cm (< 3rd percentile), respectively. Her disease remains active, evidenced by her recent endoscopic evaluation showing shallow ileal ulcers, indicating ongoing disease without remission. The patient is currently undergoing evaluation for hematopoietic stem cell transplantation (HSCT).

## 3 Discussion and conclusions

In comparison to polygenic causes in older pediatric IBD, monogenic defects play a more important role in VEO-IBD. These genetic defects have been found to influence the intestinal immune system via different mechanisms, such as disruption of the intestinal epithelial barrier (e.g., tetratricopeptide repeat domain 7A deficiency), dysfunction of phagocytes (e.g., chronic granulomatous disease), and induction of hyperinflammation through defects in the immune inhibitory mechanisms (e.g., defects in IL-10 signaling).^[6,9]^ Children with VEO-IBD most commonly have isolated colonic disease at presentation, and the disease phenotype (including macroscopic and microscopic features) and locations may change over time, which makes it challenging to classify VEO-IBD as UC or CD initially. Approximately one quarter of patients with an initial diagnosis of UC or inflammatory bowel disease-unclassified were reclassified as CD at older ages due to newly developed evidences. The unique disease course of VEO-IBD also influences the operative strategies and ileal pouch-anal anastomosis is suggested to be delayed in children with very early-onset colitis.^[3,10]^

Over the last decade VEO-IBD has become increasingly more apparent that identification of an underlying defect can lead to targeted therapy, and in some cases life-saving approaches. These children should therefore undergo a comprehensive immunologic evaluation, and genetic sequencing may be warranted as well.^[9,11]^

Interleukin-10 receptor mutations were first identified to lead to infantile onset IBD by Glocker et al. The authors showed that these mutations can abrogate IL-10 induced signal transducer and activator of transcription 3 signaling and result in deficient signal transducer and activator of transcription 3 phosphorylation as well as augmentation of tumor necrosis factor-α and other proinflammatory cytokine (e.g. IL-6, IL-1, etc.).^[7]^

Patients with IL-10R mutations usually present with refractory enterocolitis and severe perianal disease such as perianal abscess, fissures, and fistulas in infancy.^[8]^ They may also have extraintestinal manifestations such as oral ulcers, eczema, folliculitis, and arthritis and higher risk of developing B-cell lymphoma.^[12]^ Conventional immunosuppressive therapies with corticosteroids, mesalazine, azathioprine, or even tumor necrosis factor alpha inhibitor may only alleviate the symptoms but do not induce remission or long-term improvement in patients with IL-10R mutations. HSCT is the current curative therapy.^[13,14]^

Delay in diagnosis for patients with IL-10R mutations who do not receive proper therapies, are likely to receive surgical interventions including partial or subtotal colectomy or diverting stoma formation.^[15]^ However, treatment with HSCT can cure this disease and prevent catastrophic malignant transformation to large B cell lymphoma. Therefore, allogenic HSCT is critical for these children.

**Figure 4:**
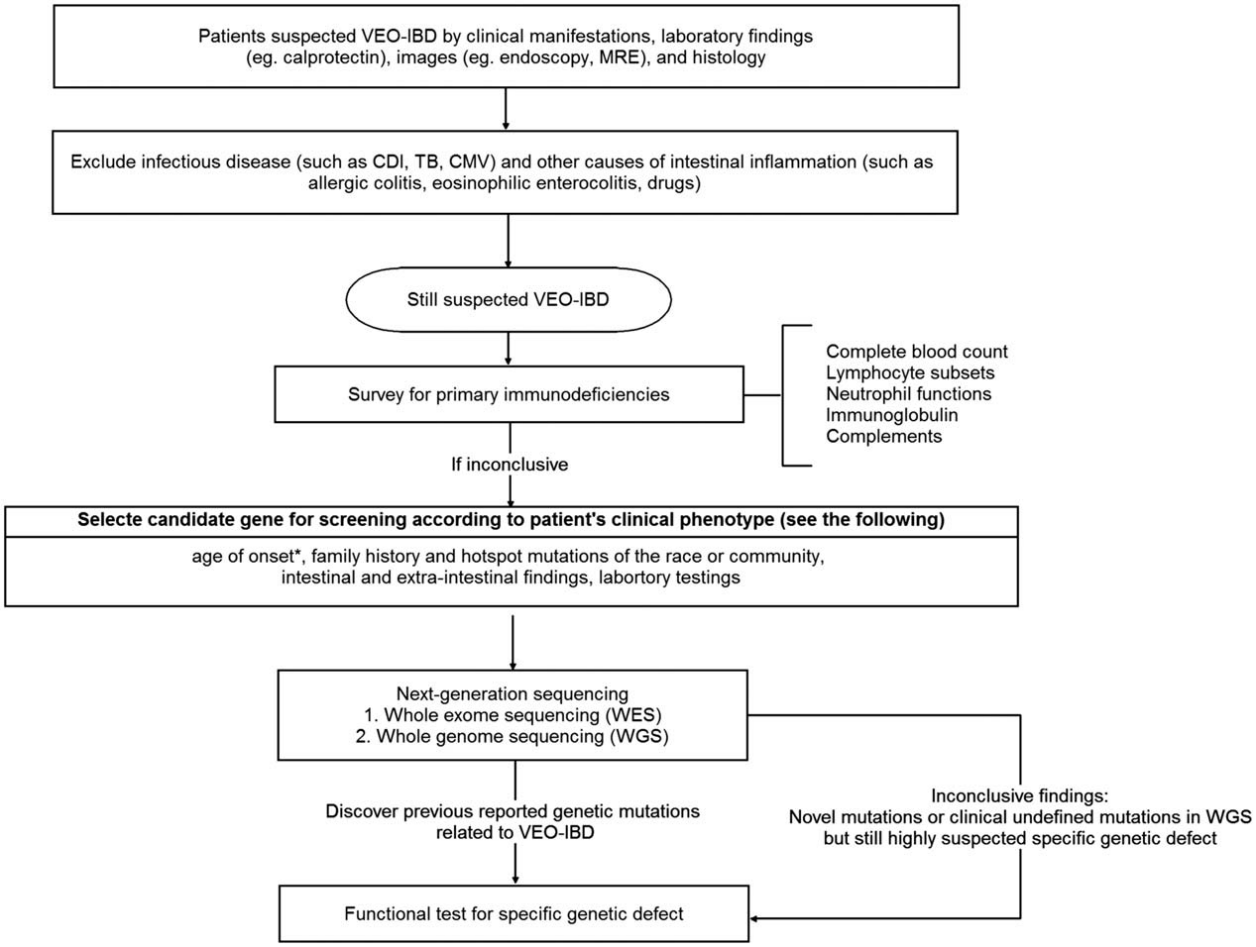
The recommended diagnostic algorithm of patient suspected VEO-IBD. VEO-IBD = very-early-onset infantile in!ammatory bowel disease, CDI = *Clostridium difficile* infection, MRE = magnetic resonance enterography, TB = tuberculosis, CMV = cytomegalovirus. ^∗^Older children with severe intestinal disease refractory to conventional treatment should also be considered to have Targeted Next Generation Sequencing screening.

In conclusion, our case highlights the importance of early detection of underlying causes of VEO-IBD, which can prevent patients from undergoing unnecessary surgeries and effects of ineffective medical therapies. The diagnostic algorithm in Figure 4 can guide clinicians in their diagnostic approach.^[9]^ Furthermore, infantile-IBD patients with complex perianal disease, intractable early onset enterocolitis and extraintestinal manifestations including oral ulcers and skin folliculitis, should undergo genetic sequencing to identify IL10 and IL10R genes mutations, and function of IL-10 signaling should also be assessed. HSCT can be curative and life-saving.

## Author contributions

**Conceptualization:** Hua-Hsi Hung, Hung-Chang Lee, Chun-Yan Yeung, Nien-Lu Wang, Tzu-Yin Tang, Harland S. Winter, Judith R. Kelsen, Chuen-Bin Jiang.

**Data curation:** Tzu-Yin Tang, Harland S. Winter, Judith R. Kelsen.

**Formal analysis:** Harland S. Winter, Judith R. Kelsen.

**Methodology:** Chun-Yan Yeung, Chuen-Bin Jiang.

**Supervision:** Hung-Chang Lee, Chun-Yan Yeung, Nien-Lu Wang, Tzu-Yin Tang, Harland S. Winter, Judith R. Kelsen. Chuen-Bin Jiang.

**Writing – original draft:** Hua-Hsi Hung.

**Writing – review & editing:** Hua-Hsi Hung, Hung-Chang Lee, Chun-Yan Yeung, Tzu-Yin Tang, Harland S. Winter, Judith R. Kelsen, Chuen-Bin Jiang.
